# Is There a Therapeutic Role for Selenium in Alpha-1 Antitrypsin Deficiency?

**DOI:** 10.3390/nu5030758

**Published:** 2013-03-11

**Authors:** Catherine M. Greene, Roohi Chhabra, Noel G. McElvaney

**Affiliations:** Respiratory Research Division, Department of Medicine, Royal College of Surgeons in Ireland, Beaumont Hospital, Dublin 9, Ireland; E-Mails: roohichhabra@physicians.ie (R.C.); gmcelvaney@rcsi.ie (N.G.M.)

**Keywords:** selenium, selenoprotein S, alpha-1 antitrypsin deficiency, chronic obstructive pulmonary disease

## Abstract

Selenium is an essential trace mineral of fundamental importance to human health. Much of its beneficial influence is attributed to its presence within selenoproteins, a group of proteins containing the rare amino acid selenocysteine. There are 25 known human selenoproteins including glutathione peroxidases, thioredoxin reductases and selenoproteins. Selenoprotein S (SEPS1) is an endoplasmic reticulum (ER) resident selenoprotein involved in the removal of misfolded proteins from the ER. SEPS1 expression can be induced by ER stress, an event that is associated with conformational disorders and occurs due to accumulation of misfolded proteins within the ER. Alpha-1 antitrypsin (AAT) deficiency, also known as genetic emphysema, is a conformational disorder in which the roles of ER stress, SEPS1 and selenium have been investigated. SEPS1 can relieve ER stress in an *in vitro* model of AAT deficiency by reducing levels of active ATF6 and inhibiting *grp78* promoter- and NFκB activity; some of these effects are enhanced in the presence of selenium supplementation. Other studies examining the molecular mechanisms by which selenium mediates its anti-inflammatory effects have identified a role for prostaglandin 15d-PGJ_2_ in targeting NFκB and PPARγ. Together these ER stress-relieving and anti-inflammatory properties suggest a therapeutic potential for selenium supplementation in genetic emphysema.

## 1. Introduction

Selenium (Se) is a dietary trace element required for many aspects of human health. Deficiency in selenium is associated with a range of morbidities, such as immunosuppression, viral infections, hypothyroidism and cardiovascular disease among many others. Se is essential due to its requirement by the 21st amino acid, selenocysteine, which is present in selenoproteins [1]. This family of proteins includes glutathione peroxidases, thioredoxin reductases, iodothyronine deiodinases and other selenoproteins such as selenoprotein S (SEPS1). 

Alpha-1 antitrypsin deficiency (AATD) is a genetic disorder associated with lung and liver manifestations [2]. The liver disease arises due to accumulation of a misfolded variant of the antiprotease AAT in the endoplasmic reticulum (ER) of liver cells. Thus lower levels of circulating AAT are available to protect the lungs from proteolytic damage leading to early-onset emphysema. The “Z” variant is responsible for >95% cases of AATD. Misfolded ZAAT is also expressed by cells in the lung and may contribute to the pulmonary inflammatory manifestations of the disorder. Little is known regarding the specific factors that predispose ZAATD individuals to lung or liver disease however environmental or idiosyncratic differences at the genetic level are likely to contribute. Moreover, the effects of specific modifier genes on disease severity and outcome have not been studied in detail. Selenoprotein S is a candidate modifier gene for ZAATD. SEPS1 is an ER protein involved in the ER stress response and degradation of misfolded proteins, such as ZAAT; its activity depends on a key selenocysteine residue in its active site. 

## 2. Selenium and the Selenoproteome

Selenium is an essential trace mineral required for mammalian immune function and anti-oxidant activity [3]. It enters the food chain through plants, which take it up from the soil. Severe selenium deficiency is associated with a number of human diseases including the cardiopathy Keshan disease and Kashin-Beck disease, a form of osteoarthritis, and is geographically linked with volcanic regions and other areas where the selenium soil content is particularly low. Less-overt selenium deficiency can have adverse consequences for disease susceptibility and maintenance of optimal human health. Compromised immune function, susceptibility to viral infection, impaired fertility, depression, cardiovascular disease, cancer, inflammatory disorders such as arthritis and dysfunctional thyroid hormone metabolism have all been linked to selenium deficiency [3,4,5,6,7,8,9,10]. 

The human genome encodes at least 25 selenoproteins [11]. These are proteins containing the rare 21st amino acid selenocysteine (Sec), usually as a key residue in the active site of these proteins. The insertion of selenocysteine into selenoproteins occurs in a peculiar way, requiring the UGA codon, which also serves as a stop codon. Other factors required for this process include a selenocysteine insertion sequence (SECIS), Sec-tRNA and specific RNA binding proteins (SBP2, L30, SECp43 and soluble liver antigen) [12]. The gene *Trsp* encodes for Sec-tRNA, which is involved in translation of all selenoproteins. Most known selenoproteins have cysteine-containing homologues however these usually have poorer activity as the Sec residue is commonly located in a selenoprotein’s active site [13]. 

### Anti-Inflammatory Properties of Selenium

**Figure 1:**
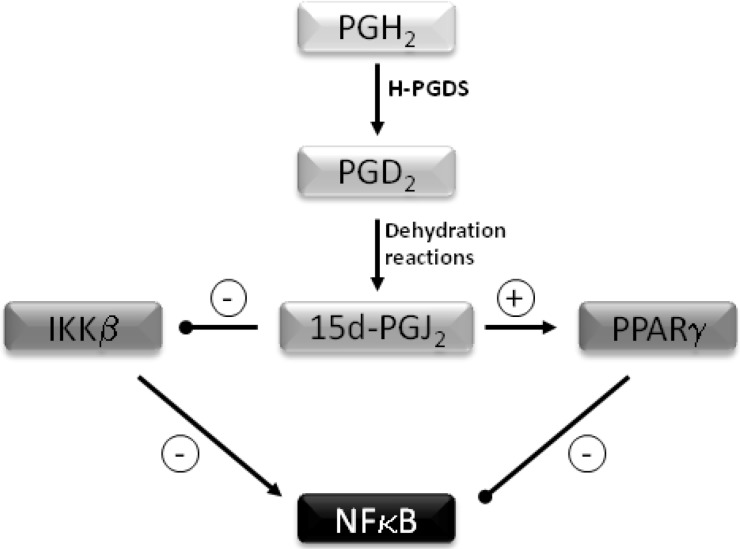
Inhibition of NFκB by 15 deoxy-PGJ_2_. Prostaglandin D_2_ synthase (H-PGDS) converts Prostaglandin (PG) H_2_ to PGD_2_. Two spontaneous dehydration reactions occur to form 15-deoxy-Δ^12,14^–prostaglandin J_2_ (15d-PGJ_2_). This inhibits IKKβ and prevents activation of NFκB. 15d-PGJ_2_ binds and activates PPARγ leading to further inhibiton of NFκB.

Selenium’s antioxidant role is mediated via glutathione peroxidases, which can reduce hydrogen peroxide and phospholipid hydroperoxides to harmless substances such as water and alcohol. In addition to its antioxidant role, Se also has anti-inflammatory properties. These are mediated largely via two mechanisms. Firstly selenium supplementation leads to enhanced formation of 15-deoxy-Δ^12,14^–prostaglandin J_2_ (15d-PGJ_2_) in macrophages via arachidonic acid oxidation by COX1 [14]. Following the conversion of prostaglandin (PG) H_2_ to PGD_2_ by hematopoietic prostaglandin D_2_ synthase (H-PGDS), two spontaneous non-enzymatic dehydration reactions occur to form 15d-PGJ_2 _ (Figure 1). This PG inhibits the IκB kinase (IKK) β subunit of the IKK complex by forming a Michael adduct with Cys-179, thereby modulating its activity and preventing activation of the proinflammatory transcription factor NFκB (nuclear factor kappa-light-chain-enhancer of activated B cells). This results in inhibition of the expression of pro-inflammatory genes such as cyclooxygenase-2 and nitric oxide synthase [14]. The second mechanism involves binding and activation of 15d-PGJ_2_ to the PPARγ (peroxisome proliferator- activated nuclear receptor gamma), again resulting in inhibition NFκB [15,16,17,18].

## 3. Alpha-1 Antitrypsin Deficiency

The AAT gene is a 12.2 kilobase gene located on the q arm of chromosome 14 at position 14q32.1 [19]. Its protein product, alpha-1 antitrypsin, is an acute phase 52-kDa 418 amino acid glycoprotein that is abundant in serum (2 mg/mL of serum). It is produced chiefly in the liver at an average rate of 2 g per day. AAT is also produced by other cell types including monocytes, neutrophils and bronchial epithelial cells [20]. AAT functions primarily as a serine protease inhibitor, and its major cognate protease is neutrophil elastase, an enzyme responsible for the degradation of a wide range of extracellular matrix and serum proteins. AAT deficiency (AATD, also known as genetic emphysema) is a monogenic autosomal co-dominant inherited disease associated with early onset emphysema and liver disease [21]. Factors that predispose individuals with AAT deficiency to lung or liver manifestations are unknown however modifier genes are likely to be important. 

Over 100 polymorphic alleles of AAT have been identified however the Z allele in particular is associated with both liver and lung disease. 10%–15% of ZAAT homozygous individuals have clinically significant liver disease in their first 20 years of life, which can manifest as hepatitis and cholestasis in neonates; a proportion of these children progress to liver failure; currently the only treatment is liver transplantation. In adults, chronic liver disease, cirrhosis or hepatocellular carcinoma can develop in 5%–10% of ZAATD individuals. Those that do not develop liver manifestations (approximately 85%–90% of the ZAAT population) characteristically develop pulmonary disease in their third or fourth decades of life. 

The classic pulmonary pathology in ZAATD is diffuse lower zone emphysema, as opposed to centrilobular emphysema seen with cigarette smoking. About 75%–85% patients develop chronic obstructive pulmonary disease. Apart from emphysema and airflow obstruction, AATD is also associated with chronic bronchitis and bronchiectasis. 

The major goal of AATD management involves preventing or slowing the progression of lung disease. Decreasing any pro-inflammatory stimuli such as smoking or respiratory tract infection helps to facilitate this aim. Interestingly, smoking cessation has been found to have the greatest impact on survival of emphysema patients as compared to any other treatment. Augmentation or replacement of the deficient protein is also possible. AAT has been purified from plasma of healthy individuals and has been delivered intravenously since 1987. It is given at a dose of 60 mg/kg body weight weekly. This dose is sufficient to raise serum AAT levels above the protective threshold of 11 μmol/L throughout therapy duration. 

### 3.1. Molecular Basis of AATD

AATD associated with the Z allele results in the accumulation of ZAAT within hepatocytes and other AAT-producing cells. The ZAAT protein differs from the normal M variant by a single amino acid substitution (Glu342→Lys) [22]. This mutation results in the disruption of an important salt bridge within the AAT protein affecting its secondary structure and causing it to adopt an aberrant conformation. As a result, the protein accumulates in the endoplasmic reticulum (ER) of hepatocytes and other AAT-producing cells [23]. ZAATD is classed amongst a group of disorders referred to as “conformational disorders”. Other conformational disorders include cystic fibrosis, Alzheimer’s, Parkinson’s and Huntington’s diseases, each of which is associated with intracellular accumulation of misfolded proteins, ER stress or an aberrant inflammatory response. Retention of ZAAT in the ER creates two problems. Its accumulation induces hepatocyte cell damage and functional ZAAT is released from the liver in only small quantities (<0.3 mg/mL serum). Consequently, low serum levels of ZAAT provide a reduced antiprotease capacity in the lung, leading to emphysema.

At a cellular level the consequences of ZAAT ER retention can include impaired secretion of ZAAT and activation of ER stress responses. Identifying factors with the potential to modulate these aberrant signalling pathways and/or facilitate ZAAT secretion has important therapeutic implications for ZAATD. Selenoprotein S (SEPS1) represents one such factor [24,25]. SEPS1 resides in the ER membrane where it processes and removes misfolded proteins from the ER to the cytosol.

### 3.2. ZAAT and Inflammation

Basally or following stimulation with a proinflammatory agonist such as lipopolysaccharide, peripheral blood monocytes from ZAAT deficient individuals secrete more cytokines than monocytes from non-AATD individuals [26]. NFκB is implicated in this response, which in this context involves activation by the kinase Akt. Thus the lung inflammation in ZAAT deficiency is not only due to inadequate AAT levels in the lumen of the lung but also due to exaggerated inflammatory responses by circulating blood cells and possibly lung resident macrophages and airway epithelial cells. 

## 4. Endoplasmic Reticulum Stress

Newly synthesised proteins destined for secretion or insertion into the membrane are transported into the endoplasmic reticulum (ER) lumen, where they are folded and assembled. ER homoeostasis is crucial for normal cell survival and function. Stress on the ER can be induced *in vitro* by a variety of methods including glucose starvation, inhibition of protein glycosylation or depletion of calcium from the ER lumen, and is characterised by a number of cellular responses [27,28,29]: Decreased protein synthesis, the unfolded protein response (UPR), the ER overload response (EOR), apoptosis, and autophagy. All of these stress responses can be induced by ER accumulation of ZAAT [27,30,31,32,33] and have been reviewed extensively [34,35,36,37]. ER-associated degradation (ERAD) is also activated in response to ZAAT expression and may be linked to aberrant SEPS1 expression and/or activity in ZAATD.

## 5. ERAD and Selenoprotein S

There is a quality control mechanism in the ER that discriminates correctly folded proteins from misfolded polypeptides and determines their fate. ERAD, the term used to describe this mechanism, involves the retro-translocation of terminally misfolded proteins from the ER into the cytosol where they are degraded by cytoplasmic proteasomes. EDEM I, a soluble ER lumen protein, is known to associate with misfolded AAT and accelerate its degradation. EDEM I is closely related to class I mannosidases but has no enzymatic activity. Its role in degradation of misfolded AAT can be enhanced by overexpression of the ER processing alpha 1,2-mannosidase ER Man I [33].

Selenoprotein S (alternatively known as SEPS1, SELS, SELENOS, VIMP or TANIS in the mouse) is a type II ER transmembrane protein involved in the ER stress response [38,39,40]. Like EDEM 1 it processes and removes misfolded proteins from the ER to the cytosol where they are polyubiquitinated and degraded by the proteosome [41]. ZAAT is known to undergo processing by this mechanism however the role of SEPS1 in ERAD of ZAAT has not been demonstrated. 

The SEPS1 gene is located on chromosome 15q26.3—a region associated with the conformational diseases diabetes and Alzheimer’s disease and it is a strong functional and positional candidate gene for various inflammation-related disorders. Genetic correlation studies have shown that the proinflammatory cytokines IL-1β, TNFα and IL-6 may be affected by a gene that jointly influences their expression. Curran *et al.* [24] genotyped the SEPS1 locus for a number of polymorphisms and identified a key single nucleotide polymorphism (SNP) at position −105 which showed significant correlation to proinflammatory cytokine plasma levels. This group demonstrated overwhelming support for a role of the SEPS1 −105G/A SNP as a functional variant for these proinflammatory biomarkers. This is of particular interest in AATD research given that an aberrant inflammatory response is a probable factor determining the severity and outcome of disease associated with ZAATD.

Interestingly the −105G/A promoter SNP has also been shown to have a key role in regulating SEPS1 expression during ER stress. The SEPS1 promoter is GC-rich and contains one putative and one conserved ER stress response element II (ERSE), a consensus-binding site for transcription factors regulating ER stress responses. ERSEs are involved in the stress inducibility of ER proteins which assist protein folding in the ER and promote cell survival under stress conditions. The −105 G/A SNP in SEPS1 is located in the putative ERSEII (attggccGgggaccacg) *vs.* (attggccAgggaccacg) for the G and A alleles, respectively. The A allele confers lower promoter activity in response to ER stress than the G allele and is associated with higher cytokine levels. Thus the phenotypes associated with the SEPS1 −105A allele (*i.e.*, low SEPS1 expression) are decreased SEPS1 expression and ERAD function and increased cytokine production [12]. Although there have been no studies to determine the frequency of the −105A SEPS1 SNP in a ZAATD population other studies have attempted to define the role of SEPS1 in ZAATD *in vitro.* This work determined the effects of SEPS1 overexpression and ablation on ZAAT-induced ER stress responses and inflammation, and characterised the effect of selenium supplementation on SEPS1 function [42].

## 6. SEPS1 and ZAAT

Selenoprotein S is known to help stressed cells by enhancing their ability to cope with the burden of misfolded proteins such as ZAAT. In order for SEPS1 to function correctly is requires selenium. Kelly *et al.* [42] investigated if adding selenium to cells can improve the way SEPS1 functions and determined what happens if SEPS1 is not present in cells. They examined three responses that are affected in liver and/or lung cells due to ZAAT accumulation and tested the effect of selenium and different SEPS1 levels on each of these three responses. 

The effect of SEPS1 on the UPR component of ER stress was investigated by measuring *grp78* promoter activity (a UPR upregulated gene) and ATF6 activation in HepG2 cells transfected with an empty vector or a ZAAT transgene. Cells that were transfected with the empty vector and treated with the ER agonist tunicamycin had significantly increased *grp78* promoter activity compared to cells transfected with the empty vector alone. Expression of ZAAT induced a similar effect. In the same cells overexpression of SEPS1 significantly decreased *grp78* promoter activity. Both tunicamycin and ZAAT also independently led to activation of the UPR-sensitive transcription factor ATF6 whilst overexpression of SEPS1 in these cells inhibited the effect.

NFκB is a pro-inflammatory transcription factor activated in ER stress. The effect of SEPS1 expression on NFκB activation was examined in HepG2 cells in response to ZAAT or IL-1β, another NFκB activator. HepG2 cells were co-transfected with an empty vector or a plasmid carrying the ZAAT transgene and an (NFκB)_5_—luciferase reporter construct. Overexpression of SEPS1 resulted in significantly reduced NFκB activation induced by ZAAT or IL-1β.

This group also investigated whether selenium supplementation could enhance the activity of SEPS1 by measuring glutathione peroxidase (GPX) activity as a surrogate marker. GPX is a selenoprotein, its activity is enhanced when it contains Sec rather than cysteine in its active site. HepG2 cells were grown in seleno-DL-methionine-supplemented medium or routine serum-containing medium. GPX activity was higher in cells grown in the presence of selenium, thereby indicating enhanced selenoprotein activity in these cells. In addition, SEPS1 protein expression was also assessed in selenium deficient and selenium sufficient conditions. It was found that addition of 40 nM to 150 μM selenium into the culture medium led to increased SEPS1 protein expression. 

Finally the anti-inflammatory effects of selenium on 15d-PGJ_2_ was evaluated. When HepG2 cells were grown in the absence or presence of seleno-DL-methionine for 48 h there was increased concentration of 15d-PGJ_2_ in the selenium supplemented cells. In view of these results selenium supplementation may have therapeutic potential for ZAATD.

### Selenium Status in ZAATD

The dietary selenium intake in many countries in Europe and other parts of the world is still below the range recommended by the health regulatory bodies [43]. The selenium intake in Europe is considerably lower due to low selenium concentrations in the soil. The recommended range for selenium intake in Europe is therefore higher, the UK reference nutrient intake being 75 μg/day for men and 60 μg/day for women. The current selenium recommendation in the United States is 55 μg/day. This level is based on optimising plasma GPX activity. Suboptimal selenium status may lead to suboptimal functioning of SEPS1, and may also increase susceptibility to various chronic disorders. Apart from one small study that measured serum selenium levels in 12 age- and sex-matched AAT deficient verus AAT sufficient individuals little is known regarding the selenium status of ZAATD individuals [42]. Although no major differences were found between the two groups, the mean serum selenium levels were found to be lower than the optimal range for serum GPX activity (70–90 μg/L) in 75% of the ZAATD patients. 

## 7. Chronic Obstructive Pulmonary Disease

Chronic obstructive pulmonary disease (COPD) is the fourth leading cause of death worldwide [44]. The major risk factor for the development of COPD is cigarette smoke however other environmental factors such as heavy exposure to occupational dusts and chemicals (vapours, irritants, and fumes) and indoor/outdoor air pollution are also important. Genetic predisposition is also responsible for COPD: to date AATD is the only proven genetic cause of COPD. Approximately 1% of patients with COPD are AAT deficient [45] but this is likely to be an underestimation due to the lack of awareness and underdiagnosis of ZAATD. Non-AATD COPD is a polygenic disease. For example the alpha-nicotinic acetylcholine receptor (CHRNA 3/5) and hedgehog interacting protein (HHIP) loci, identified in a genome-wide association study of COPD, are thought to contribute to the risk of COPD [46].

### 7.1. COPD and ER Stress

There have been various reports implicating ER stress, and the UPR in particular, in COPD pathogenesis. A series of reviews cover this topic in detail [47,48,49,50]. Oxidative stress in the lung occurs as a consequence of cigarette smoking and interferes with protein folding in the ER leading to ER stress. For example, *in vitro* normal human bronchial epithelial cells treated with cigarette smoke extract (CSE) show a dynamic change in PERK-eIF2α pathway activity [51] and increases in the ER stress response through activating transcription factor 4 (ATF4)-mediated induction of C/EBP homologous protein (CHOP). Primary small airway epithelial cells and macrophages respond similarly to CSE [52], as do the alveolar epithelial cell line A549. These cells response to CSE with accumulation of damaged proteins that are inefficiently degraded by the proteasome [53]. Interestingly although acute exposure to cigarette smoke directly impairs proteasome activity in the lungs of mice and in human epithelial cells it does not affect proteasome expression [54].

In experimental animals exposure to cigarette smoke can induce up to 4-fold increases in phosphorylation of eIF2α and activated ATF6 [55]. Systemic administration of acrolein a component of inhaled cigarette smoke can induce ER stress proteins in rat lung tissue with significant up regulation of ATF4, CHOP and GADD34 expression [56]. Likewise in smokers with COPD a considerable accumulation of acrolein-protein adducts in the inflammatory, airway and vascular cells is evident [56]. Impaired Nrf2 signalling has been implicated in ER stress responses in the lungs of patients with COPD and in cigarette smoke-exposed mice [57]. Min *et al.* recently demonstrated that both acute and subchronic cigarette smoke exposure can modulate ER stress in murine lungs and verified that changes in ER stress do occur with increasing severity of emphysema in COPD [58].

Whether the ER stress-relieving properties of selenium as shown for AATD *in vitro,* can extend to relieve ER stress induced by CS has not been studied however, there is evidence to suggest that selenium supplementation could have beneficial effects in COPD.

### 7.2. Selenium and COPD

Several dietary antioxidants have been positively associated with lung function in the healthy, general population. Although some studies suggest that there is no effect of selenium on COPD [59,60], a number of well controlled, strongly powered studies have found evidence that selenium can have therapeutic benefit for chronic obstructive lung disease. For example in COPD patients with acute upper respiratory tract infections, co-administration of selenium with zinc, vitamin C and *Echinacea purpurea* resulted in significantly less severe and shorter exacerbation episodes as compared with placebo [61]. In another randomized double-blinded controlled trial intravenous administration of Se with zinc and manganese to COPD patients on mechanical ventilation significantly reduced the period these patients spent on ventilation [62]. Another report concluded that maintaining higher serum concentrations of dietary antioxidant vitamins and selenium is potentially beneficial to lung health as measured by higher levels of FEV1 [63]. These findings concurred with a study of 18,162 subjects examining the separate and joint effects of vitamin C, vitamin E, beta-carotene and selenium intake data, wherein serum selenium had a strong positive association with FEV1 in smokers [64]. In an unrelated but similar study lower levels of selenium in plasma were found in COPD patients, being more evident in those with very low levels of arterial oxygen pressure [65]. 

## 8. Conclusions

Selenium supplementation has potential to be investigated as a therapeutic option for ZAAT deficiency and COPD due to its crucial role in optimal selenoprotein function. In ZAAT deficiency, the accumulation of misfolded protein in the ER results in ER stress and activation of various stress responses including UPR, EOR and inflammation. Although occurring by different mechanisms ER stress is also an important feature of the lung disease in COPD. Hence, there is scope for further research in order to gain greater understanding of the role of selenium and SEPS1 in these diseases. 
